# The Relative Apical Sparing Strain Pattern in Severe Aortic Valve Stenosis: A Marker of Adverse Cardiac Remodeling

**DOI:** 10.3390/jpm14070707

**Published:** 2024-07-01

**Authors:** Dovilė Ramanauskaitė, Giedrė Balčiūnaitė, Darius Palionis, Justinas Besusparis, Edvardas Žurauskas, Vilius Janušauskas, Aleksejus Zorinas, Nomeda Valevičienė, Peter Sogaard, Sigita Glaveckaitė

**Affiliations:** 1 Clinic of Cardiovascular Diseases, Institute of Clinical Medicine, Faculty of Medicine, Vilnius University, Santariškių Str. 2, LT-08410 Vilnius, Lithuania; 2 Department of Radiology, Nuclear Medicine and Medical Physics, Institute of Biomedical Sciences, Faculty of Medicine, Vilnius University, Santariškių Str. 2, LT-08410 Vilnius, Lithuania; 3 Department of Pathology, Forensic Medicine and Pharmacology, Institute of Biomedical Sciences, Faculty of Medicine, Vilnius University, P. Baublio Str. 5, LT-08406 Vilnius, Lithuania; 4 Departament of Cardiology, Faculty of Medicine, Department of Clinical Medicine, Aalborg University Hospital, Hobrovej 18-22, 9100 Aalborg, Denmark

**Keywords:** aortic stenosis, cardiac amyloidosis, speckle-tracking echocardiography, aortic valve replacement, relative apical sparing

## Abstract

Background: The presence of a relative apical sparing (RAS) echocardiographic strain pattern raises a suspicion of underlying cardiac amyloidosis (CA). However, it is also increasingly observed in patients with aortic stenosis (AS). We aimed to evaluate the prevalence, dynamics, and clinical characteristics of the RAS strain pattern in severe AS patients who had been referred for surgical aortic valve replacement (SAVR). Methods: A total of 77 patients with severe AS and without CA were included with a mean age of 70 (62–73) years, 58% female, a mean aortic valve area index of 0.45 ± 0.1 cm^2^/m^2^, and a mean gradient of 54.9 (45–70) mmHg. Results: An RAS strain pattern was detected in 14 (18%) patients. RAS-positive patients had a significantly higher LV mass index (125 ± 28 g/m^2^ vs. 91 ± 32, *p* = 0.001), a lower LV ejection fraction (62 ± 12 vs. 68 ± 13, *p* = 0.040), and lower global longitudinal strain (–14.9 ± 3 vs. –18.7 ± 5%, *p* = 0.002). RAS strain pattern-positive patients also had higher B-type natriuretic peptide (409 (161–961) vs. 119 (66–245) pg/L, *p* = 0.032) and high-sensitivity troponin I (15 (13–29) vs. 9 (5–18) pg/L, *p* = 0.026) levels. Detection of an RAS strain pattern was strongly associated with increased LV mass index (OR 1.03, 95% CI 1.01–1.06, *p* < 0.001). The RAS strain pattern had resolved in all patients by 3 months after SAVR. Conclusions: Our findings suggest that the RAS strain pattern can be present in patients with severe AS without evidence of CA. The presence of an RAS strain pattern is associated with adverse LV remodeling, and it resolves after SAVR.

## 1. Introduction

Cardiac amyloidosis (CA) is caused by an abnormal build-up of amyloid fibrils in the left ventricle (LV), leading to myocardial thickening, stiffness, and diastolic dysfunction. Over time, the disruption of LV loading conditions due to amyloid deposition leads to the development of restrictive cardiomyopathy, which in turn manifests as congestive heart failure. Transthyretin cardiac amyloidosis (ATTR-CA) has been increasingly recognized in patients with degenerative aortic stenosis (AS) [1,2,3]. As survival rates improve and the number of older patients rises, the prevalence of both ATTR-CA and AS also increases. Consequently, a growing number of studies have investigated their coexistence [4,5,6,7]. Existing data reveal that, within the group of patients with severe AS who undergo transcatheter aortic valve replacement (TAVI), one in eight patients also have CA [8,9]. Recognizing concurrent ATTR-CA in patients with AS can be very challenging due to the similar clinical and echocardiographic characteristics shared by both diseases.

According to the latest guidelines, non-invasive diagnostic strategies are recommended for the diagnosis of CA, thereby reducing the use of invasive methods such as endomyocardial biopsy. Cardiovascular magnetic resonance (CMR) with late gadolinium enhancement (LGE), T1 parametric mapping, and bone scintigraphy have now become the main diagnostic tools for CA.

Despite advances in diagnostic methods, speckle-tracking echocardiography (STE) remains one of the key non-invasive and easily available methods for the assessment of AS, and global longitudinal strain (GLS) is a sensitive marker of early LV systolic dysfunction. Furthermore, GLS is also used in the screening of healthy hearts for cardiac involvement in amyloidosis [10,11]. GLS is relatively uniform throughout the LV; however, in CA, there is often variation in regional longitudinal strain, with impaired strain values measured in the basal and midventricular regions of the LV compared with those in the apex. The relative apical sparing (RAS) strain pattern comprises a ratio of apical versus mid-basal and basal longitudinal strain values, with a result above 1 for this ratio raising a suspicion of a CA diagnosis. However, some data are giving rise to doubts about the specificity of the RAS strain pattern for detecting CA in patients with AS [12].

The aim of this study was to assess the frequency and clinical characteristics of the RAS strain pattern in patients with severe AS and to determine the dynamics of the RAS strain pattern following surgical aortic valve replacement (SAVR).

## 2. Materials and Methods

### 2.1. Study Design and Population

**Figure 1:**
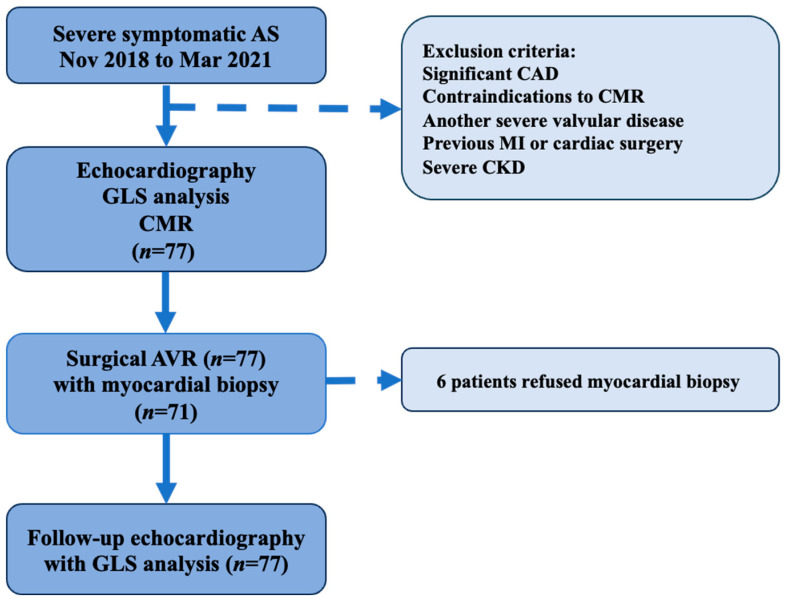
Study design flow chart. AS, aortic stenosis; AVR, aortic valve replacement; CAD, coronary artery disease; CMR, cardiovascular magnetic resonance; CKD, chronic kidney disease; GLS, global longitudinal strain; Mar, march; MI, myocardial infarction; *n*, number; Nov, November.

This prospective observational study was conducted at the Vilnius University Hospital Santaros Klinikos from November 2018 to March 2021. Patients with severe symptomatic AS who were scheduled for SAVR as per current treatment recommendations [13] were enrolled in the study. The design of the studio is shown in Figure 1. This study was approved by the local Biomedical Research Ethics Committee (Approval Number: 158200-18/9-1014-558) and was performed as part of the FIB-AS Study (NCT03585933). All participants provided written consent prior to enrollment as stated in the principles of the Declaration of Helsinki. Patients were recruited prior to a pre-operative assessment, and they underwent a clinical assessment at which their clinical history was taken and they completed the Minnesota Living with Heart Failure Questionnaire, performed a 6 min walking test (6MWT), had a blood sample collected (for hematocrit, renal function, brain natriuretic peptide (BNP), and high-sensitivity troponin I (Hs–Tn–I) measurement), and underwent both a transthoracic echocardiogram with GLS analysis and a CMR with T1 mapping.

### 2.2. Inclusion/Exclusion Criteria

The inclusion criteria for this study allowed for the recruitment of patients who were undergoing AVR for severe AS (defined as an aortic valve area (AVA) ≤ 1 cm^2^ or an AVA index ≤ 0.6 cm^2^/m^2^, as determined by echocardiography), were over 18 years old, were able to undergo a CMR scan, and gave informed consent to participate in the study.

The exclusion criteria were as follows: a history of significant coronary artery disease (CAD) (>50% lesion) or the presence of CMR-incompatible devices. All exclusion criteria are reported in Figure 1. The study data were collected and stored in a dedicated online database using REDCap 13.1.37 (Research Electronic Data Capture) [14].

### 2.3. Cardiac Imaging

#### 2.3.1. Echocardiography

Transthoracic 2D echocardiography was performed using a Vivid ultrasound system (model S70, E9, or E95) from GE Healthcare, Horten, Norway. Acquired data were stored on a dedicated workstation for post-processing analysis. Images were obtained and optimized and AS severity, LV systolic function, and diastolic function were evaluated following recommended echocardiographic guidelines [11,15]. The LV GLS was measured and processed offline using commercially available software (EchoPac 112.0.1) from GE Medical Systems, Horten, Norway [16]. GLS was acquired using the averages of regional strain curves of a 17-segment model for 2D STE. Patients with poor-quality tracking echocardiography results or aberrant curves (even after manual adjustment) were removed from the study. Echocardiographic examinations with GLS analysis were repeated 3 months and 12 months after SAVR.

#### 2.3.2. CMR Protocol

CMR scans were obtained using standard protocols on a 1.5 T Siemens Aera scanner (Erlangen, Germany) with surface coils and retrospective electrocardiography (ECG) triggering. CMR measurements, including LV volumes, mass, and ejection fractions, were assessed using commercial software (suiteHEART^®^, Neosoft, Pewaukee, WI, USA) and indexed to body surface area (BSA) in m^2^ (using the DuBois formula). Technical specifics regarding post-contrast LGE imaging, native and post-contrast T1 mapping, and measurement protocols have been published previously [17]. Due to incomplete datasets, T1-mapping parameters were measured in 67 out of 77 patients.

### 2.4. Histological Analysis

During SAVR, the surgical team obtained biopsy specimens under direct vision using a surgical scalpel. These samples were taken from the basal anteroseptum immediately after the diseased AV was removed. One intraoperative myocardial biopsy sample (mean area, 22.5 ± 12 mm^2^) was taken from each patient.

The detailed methodology is described in a previous article [17].

### 2.5. Statistical Analysis

Variables are expressed as mean ± standard deviation or median with interquartile ranges. The normality of the distribution was assessed by Shapiro–Wilk normality tests. Categorical variables are expressed as frequencies and percentages and were compared by a Chi-squared test. For continuous variables, differences between two groups were assessed using unpaired Student’s *t*-tests and Mann–Whitney U tests. To evaluate differences among three related samples, repeated measures ANOVA and Friedman tests were employed to identify statistically significant differences. Receiver operating characteristic (ROC) curves were generated for the LV mass index to determine cut-off values. Statistical analysis was conducted using R software (version 4.1.2), and statistical significance was defined as a 2-sided *p*-value < 0.05 [18].

### 2.6. Intra-Observer and Inter-Observer Variability Analysis

Variability analysis revealed good intra- and inter-observer reproducibility for post-contrast T1 and GLS measures. The intra-observer and inter-observer reproducibility values and 95% CIs were as follows: native T1, 0.958 with 95% CI 0.91–0.98 and 0.945 with 95% CI 0.88–0.97, respectively; post-contrast T1, 0.97 with 95% CI 0.94–0.99 and 0.987 with 95% CI 0.9–0.99, respectively; and GLS, 0.981 with 95% CI 0.96–0.99 and 0.969 with 95% CI 0.93–0.98, respectively.

## 3. Results

### 3.1. Study Cohort Data

A total of 77 patients with severe AS were included in the study (mean age, 70 (age range, 62–73 years); 58% female; mean aortic valve area (AVA) index, 0.45 ± 0.1 cm^2^/m^2^; mean peak aortic valve (AV) velocity, 4.9 ± 0.6 m/s; mean AV gradient, 54.9 (45–70) mmHg). The majority of patients were symptomatic (62 (81%) of them were classified in the NYHA ≥ II functional class). The plasma Hs-Tn-I concentration was 10 (5–18) pg/L and the mean BNP was 142 (67–362) pg/L. No low voltage criteria on the ECG were observed. The mean LVEF value was 69.6 (61–75), and 10% of patients showed reduced LVEF (<50%). Most patients were classified as low surgical risk, with STS-PROM and EuroScore II values below 2%. Significant CAD, renal dysfunction, and other valvular abnormalities were the main reasons for non-eligibility. All 77 patients underwent SAVR, and most patients (92%) received a biological AV prosthesis. In addition to AVR, aortic surgery was performed in 3% of patients. The patients’ clinical, imaging, and histological characteristics are summarized in Table 1.

### 3.2. Data Comparison between Patients with and without an RAS Strain Pattern

As shown in Table 1, the mean GLS was −18 ± 5% (ranging from −3 to −31%), and reduced GLS (>−15%) was observed in 21% of study patients. A pre-operative RAS strain pattern was found in 14 (18%) out of 77 patients. We compared the clinical and imaging characteristics of patients with and without a detectable RAS strain pattern. Patients with an RAS strain pattern had more advanced AS, with a higher AV peak velocity (*p* = 0.005) and a higher mean AV gradient (*p* = 0.013), as compared with patients not having an RAS strain pattern. Additionally, RAS strain pattern-positive patients showed evidence of more advanced LV remodeling with the following findings: thicker interventricular septum (IVS) (*p* = 0.004), larger LV diastolic diameter (*p* = 0.006), and larger LV mass index (*p* = 0.001) as assessed by CMR. Furthermore, RAS strain pattern-positive patients had worse LV systolic function, with significantly reduced GLS (*p* = 0.002) and lower LVEF (*p* = 0.040), than did RAS strain pattern-negative patients. Reduced LVEF (<50%) was only observed in patients with an RAS-type GLS pattern. In addition, laboratory analyses revealed evidence of myocardial injury in RAS-positive patients, as they had higher serum levels of BNP (*p* = 0.032) and Hs-Tn-I (*p* = 0.026) than did RAS strain pattern-negative patients. Overall, the RAS strain pattern-positive patients demonstrated more advanced LV remodeling and evidence of heart failure than did RAS strain pattern-negative patients.

When analyzing CMR T1 mapping data, we found no evidence of extracellular space expansion either in the whole study cohort or in the RAS strain pattern-positive patient group, as T1 mapping markers were not elevated (mean native T1 = 971 ± 36 ms, mean ECV = 23.4 ± 3%). Myocardial fibrosis, as assessed by LGE–CMR, was more frequently found in RAS strain pattern-positive patients than in RAS strain pattern-negative patients; however, this difference did not reach statistical significance.

**Figure 2:**
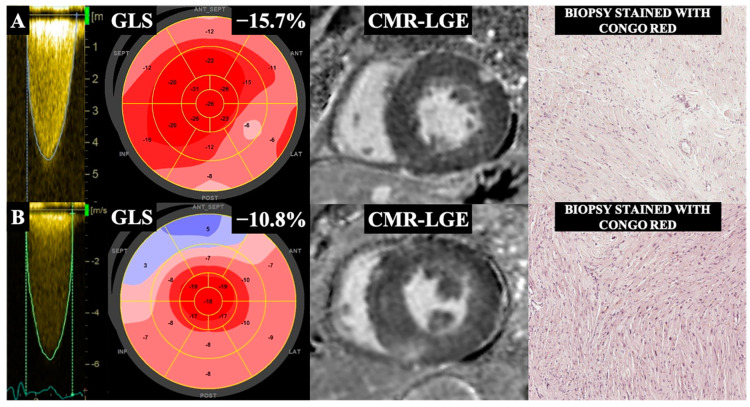
Two representative patients with severe AS, with (**B**) and without (**A**) an RAS strain pattern, showing evidence of LV remodeling. Column 1 shows continuous-wave Doppler images (maximum velocities > 4 m/s. Column 2 presents GLS analysis with a Bull’s eye map. Column 3 shows cardiovascular magnetic resonance (CMR) short-axis late gadolinium enhancement (LGE) images illustrating focal myocardial fibrosis, and Column 4 shows matching myocardial biopsies stained with Congo Red. Patient A shows reduced GLS without an RAS strain pattern, concentric LV hypertrophy, subepicardial LGE in the anterior-lateral wall, minimally expressed focal enhancement in the inferior LV–RV junction, and no pathological accumulation of amyloid. Patient B shows low GLS with an RAS strain pattern, evidence of LV hypertrophy, focal LGE in the LV–RV junctions (more pronounced in the inferior junction), and no evidence of CA.

Histological analysis of myocardial biopsies taken at the time of SAVR also revealed no evidence of amyloid deposition in any myocardial samples. We found no difference in the amount of myocardial fibrosis, as assessed histologically, between the RAS strain pattern-positive and RAS strain pattern-negative patient groups (10.8 (7–17) vs. 15.9 (8–23), *p* = 0.232, respectively). Representative examples of two RAS strain pattern-positive patients are presented in Figure 2.

**Figure 3:**
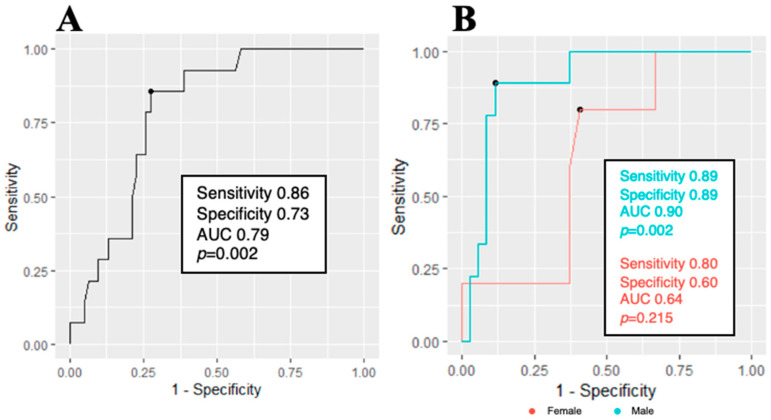
ROC curves. (**A**) shows the ability of an LV mass index cut-off value of 103.7 g/m^2^ to predict RAS strain pattern positivity. (**B**) shows data analysis by gender subgroups and a stronger relationship in the male group. AUC, area under the curve.

ROC analysis was performed to investigate the ability of LV mass index, as assessed by CMR, to predict RAS strain positivity. With an LV mass index cut-off value of greater than 103.7 g/m^2^, it was possible to predict RAS strain pattern positivity with 86% sensitivity and 73% specificity (area under curve = 0.79, Odds Ratio = 1.032, 95% CI [1.01–1.06], and *p* = 0.002; Figure 3). When analyzing the data separately by gender, this relationship was even stronger in the female group, displaying an AUC of 0.9 with both sensitivity and specificity at 0.89.

### 3.3. Follow-Up Echocardiography Data

Follow-up echocardiographic examinations conducted post-SAVR showed reverse remodeling of the LV as determined by a decrease in the IVS diameter from the baseline of 14.5 mm (range, 14.0–15.0 mm) to 11.5 mm (range, 11.0–14.0 mm) at 3 months and to 11.5 mm (range, 11.0–12.0 mm) at 12 months (*p* < 0.001). Furthermore, a significant decrease in the left atrial volume index (*p* = 0.009) and mean E/e’ (*p* = 0.004) indicated an improvement in LV loading conditions (Table 2).

An RAS strain pattern was no longer visible in any of the 14 patients at a follow-up 3 months after SAVR. Improvements in GLS were also observed 3 months after SAVR (−18.3 ± 2%), with further improvement observed at the 12-month follow-up (−19.7 ± 2%).

**Figure 4:**
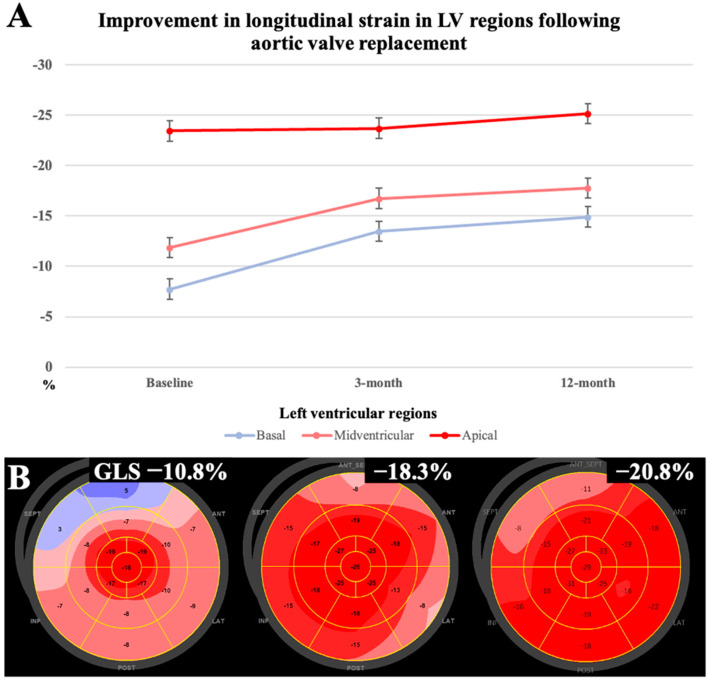
(**A**) Significant changes in the mean GLS of basal, midventricular, and apical regions (*p* < 0.001, *p* < 0.001, *p* = 0.004, respectively) relative to baseline at the 3- and 12-month follow-ups. (**B**) Example of a patient with AS exhibiting low GLS and an RAS strain pattern, with heart failure symptoms of NYHA class III and preserved LVEF at baseline, with a subsequent improvement in GLS at the 3- and 12-month follow-ups.

When analyzing longitudinal strain changes in different LV regions, we observed a significant improvement in longitudinal LV strain in the basal segment (−7.7 ± 2.1, −13.5 ± 2.0, −14.9 ± 1.9, *p* < 0.001) and the midventricular segment (−11.9 ± 2.5, −16.8 ± 1.9, −17.8 ± 1.9, *p* < 0.001) at 3- and 12-month follow-ups compared with the pre-operative assessment (Figure 4). Although the longitudinal strain in apical segments was preserved, it also showed significant changes at follow-up (−23.6 ± 2.1, −23.7 ± 1.5, −25.2 ± 1.3, *p* = 0.004).

## 4. Discussion

This is a prospective study that evaluated the prevalence and dynamics of the RAS strain pattern and its associations with other clinical parameters in surgically treated patients with low-risk but severe AS without biopsy and CMR-proven CA. The results can be summarized as follows: (1) the RAS strain pattern could be detected in up to 18% of surgically treated patients with low-risk AS who were CA negative; (2) the RAS strain pattern is reversible after SAVR; and (3) the RAS strain pattern in AS patients represents more severe AS, more advanced LV remodeling, and the presence of heart failure.

### 4.1. CA Links with AS

The RAS strain pattern is considered a classical and early “red-flag” echocardiographic feature of CA [19]. However, our data suggest that certain AS patients may also exhibit this GLS pattern without either CMR or histological evidence of an infiltrative disorder. We found that patients presenting with a relative apical GLS sparing pattern had more severe AS with a higher AV peak velocity and a higher mean AV gradient as compared with patients without a positive RAS strain pattern. Results from recent studies support our findings [12,20,21,22,23,24]. Abecasis et al. [12] analyzed 150 patients with a predominance of normal-flow, high-gradient severe AS with preserved LVEF and without CA based on histological or imaging data referred for SAVR (mean age, 73 years; interquartile range, 68–77 years; 51% female). In this study, an RAS pattern was found in 15.3% (n = 23) of patients. Moreover, there are data indicating the presence of the RAS strain pattern in other noninfiltrative cardiomyopathies. Huang et al. [20] conducted a study evaluating the RAS strain pattern in patients with LVH and revealed that 3.9% of patients with hypertrophic cardiomyopathy exhibited an RAS strain pattern.

The exact pathophysiological mechanisms behind the RAS strain pattern in cases of CA and AS remain unknown. Studies suggest that, in CA, longitudinal contraction of the basal segments deteriorates due to amyloid deposition and increased cardiomyocyte apoptosis [25,26,27]. Ternacle et al. [28] found that the extent of relative myocardial amyloid infiltration progresses from the base to the apex while increasing stiffness in these regions of the LV. Furthermore, it has been shown that the RAS strain pattern is more frequently detected in advanced stages of CA with increased myocardial mass [20,29].

Asymmetric septal hypertrophy, which is commonly detected in CA, may also contribute to the RAS strain effect [30]. Additionally, a study by Thakker et al. suggested that the RAS strain pattern can be identified by the presence of basal hypertrophy [24]. Meanwhile, unfavorable hemodynamic conditions in the LV with AS lead to stress-induced ischemia, which promotes the dysfunction of subendocardial fibers. These longitudinal subendocardial fibers are more sensitive to reduced coronary blood flow [31,32,33]. As the stress distribution is uneven in the LV, this results in impaired longitudinal contraction, which is particularly prominent in the basal region of the LV [34]. It has been found that subendocardial longitudinal strain is significantly more related to the severity of AS than are other strain measures [35,36]. The earliest decreases in GLS may even be observed with moderate AS [31]. Subsequently, as pressure overload persists, these changes gradually progress throughout the myocardium, leading to replacement fibrosis. A previous study demonstrated that myocardial segments positive for LGE were located at the base of the LV [37]. This suggests that noncontractile fibrotic tissue plays a role in longitudinal contraction.

As has been previously reported [1,2,3], AS with amyloid infiltration has been increasing in prevalence, which is an important issue due to a two-fold increase in the risk of all-cause mortality associated with AS–CA co-occurrence [6,38]. There are several possible reasons that could account for why CA was not identified in our study group. Firstly, our cohort consisted of younger patients with a median age of 70 (range, 62 to 73), 36% of whom had congenital AV disease and were considered low surgical risk. We did not include older patients who were undergoing TAVI. According to the literature, wild-type ATTR-CA is more common in the elderly population [3,5,39,40]. Published data indicate that the mean age of AS patients testing positive for ATTR-CA ranges between 75 and 88 years. The mechanism behind this age-related prevalence is unclear; however, in ATTR-CA, normal transthyretin genetic sequences suggest protein instability and altered aggregation due to aging. In a previous study of severe AS patients undergoing SAVR, ATTR amyloid was detected in 6 (4.1%) of 146 biopsies taken at surgery and confirmed by histology [41]. The prevalence increased to 5.6% when older AS patients (aged over 65) were included in the study. Secondly, due to the low AS–CA prevalence in surgical cohorts, the tested sample size could be insufficient to detect the disease. In a study by Treibel et al. [42], 146 biopsies were taken, which is twice as many as in our study group. Lastly, in comparison with SAVR patients, the prevalence of ATTR-CA AS in TAVI patients is two to three times higher, ranging between 9% and 16%. For this reason, screening for CA in TAVI-eligible patients could be the focus of future research studies at our center.

### 4.2. Consequences of AS-Induced Remodeling

Various LV-remodeling changes have been described in response to chronic pressure overload caused by AS [43]. These changes begin with compensatory hypertrophy accompanied by myocardial fibrosis, progressing to systolic dysfunction and heart failure. We suspect that the extent of remodeling may impact the RAS strain pattern presentation in AS, which would allow clinicians to identify those patients with more advanced LV alterations. An association between the GLS reduction and the size of LV hypertrophy in AS patients has been demonstrated. Anan et al. [44] showed a significantly lower degree of global longitudinal deformation in the concentric hypertrophy group compared with the normal and eccentric hypertrophy groups. This was further supported by a correlation between GLS recovery and the regression of LV hypertrophy following SAVR [39]. Furthermore, there is evidence suggesting that gender differences have significant effects on LV remodeling, which is more pronounced in males [40]. Our study results show a strong association between the increase in LV mass and the RAS strain pattern. This relationship may be attributed to more severe AS, resulting in higher hemodynamic stress and more pronounced LV hypertrophy in the RAS strain pattern group. Our ROC analysis revealed that we can effectively predict the RAS strain pattern by using a predefined LV mass index, achieving both high sensitivity and specificity. However, in our cohort, this indicator showed better predictive value in females than in males, probably due to the small number of male patients with an RAS strain pattern in this study. This shows the need for further investigation to determine gender-related thresholds for LV remodeling.

As AS worsens, the longstanding pressure overload eventually leads to reduced LV systolic function [41]. We found that LV dysfunction was more pronounced in RAS pattern-positive patients as they presented with a lower ejection fraction and reduced GLS than did RAS strain pattern-negative patients. Moreover, these patients had higher levels of BNP and Hs-Tn-I, indicating heart failure and myocardial injury. The study by Abecasis et al. [45] obtained similar results to our study, also suggesting the occurrence of an RAS strain pattern in more advanced AS. They found that patients with RAS-type GLS had more severe AS, larger indexed LV mass, a lower ejection fraction, as well as markers of myocardial damage, including higher N-terminal pro-BNP compared with patients without RAS-type GLS. Notably, they found a significantly higher prevalence of delayed enhancement on CMR in patients with RAS deformation than in those without it. Our CMR with LGE data show that focal fibrosis was affecting patients with an RAS strain pattern more frequently than those without it, but this difference did not reach statistical significance. GLS appears to be a sensitive diagnostic tool to assess the extent of LV myocardial remodeling, and the identification of an RAS strain pattern should prompt a more detailed examination of the LV to avoid fibrosis-related functional and structural abnormalities.

### 4.3. GLS as a Prognostic Marker

It is essential to identify markers of myocardial injury earlier than the onset of clinical symptoms. GLS is recognized as an important marker of subclinical LV dysfunction [46,47,48]. Moreover, prior studies showed that GLS assessment is a valuable tool for identifying patients who will benefit most from AVR [49,50]. Vollema et al. [51] found that an impaired GLS predicts an increased risk of symptom development and the need for AV intervention. These findings are further supported by a report utilizing a decrease in GLS in basal regions as a significant predictor of future SAVR in asymptomatic AS patients [52]. Furthermore, in order to evaluate patient outcomes, detection of an RAS strain pattern has been shown to improve the prediction of major adverse cardiovascular events (MACEs) in patients with LV hypertrophy [53]. Although our study did not assess patient outcomes, we assume that the subclinical evaluation of LV function using STE may prevent irreversible LV dysfunction and be a promising risk assessment method for patient outcomes. Another study [54] highlights the effectiveness of GLS in assessing the risks of AS patients, thereby encouraging its integration into current treatment guidelines. Until now, the LVEF has been the main decision-making criterion for planning SAVR in asymptomatic AS patients. The assessment of systolic function alone is considered insufficient for referring asymptomatic patients for surgical treatment [54,55,56], as it has been shown that asymptomatic (or minimally symptomatic) patients with GLS above −15.0% and preserved LVEF experienced a high risk of adverse cardiovascular outcomes [57]. In contrast, preserved GLS in AS is associated with improved LV reverse remodeling and systolic function following SAVR [54,58]. Considering that the stress caused by AV pathology can be tolerated by the LV for a long time and the reduction in the LVEF is detected only in the late stages of the disease, it is important to have a more sensitive marker to detect subclinical LV dysfunction earlier.

### 4.4. RAS Strain Pattern after SAVR

The improvement in GLS after SAVR suggests that it is the result of afterload reduction. This is supported by the previously mentioned study by Abecasis et al. [12], in which the RAS strain pattern was reversible, with only two out of the twenty-three patients with a pre-operative RAS pattern retaining a disturbed strain pattern between the third and sixth months after SAVR. Our study showed that, with reduced loading pressure, the RAS strain pattern regressed postoperatively until it had completely resolved by the third month of follow-up. As has been demonstrated by feature-tracking computed tomography, mid-basal longitudinal strain also significantly increases after TAVI [59]. In our study, along with a significant improvement in GLS in the basal and midventricular segments, increased longitudinal contraction was also observed in the apical segments (*p* = 0.004). Furthermore, a significant decrease in IVS diameter was found during follow-up, indicating reversible structural changes to the LV. A study examining reverse remodeling in patients with AS and ATTR-CA compared to patients with isolated AS post-TAVI revealed that only lone AS exhibited a significant decrease in LV hypertrophy, as evidenced by a reduction in IVS thickness [60]. Interestingly, there was no significant change in LVEF post-AVR in either group. This provides evidence for GLS as an initial and potentially reversible marker of LV remodeling.

Exploring echocardiographic parameters that do not necessarily indicate disease but offer valuable insights into cardiac function is essential. This is one of a few research studies worldwide that has systematically screened AS patients for ATTR-CA by performing multimodality imaging and histological analysis. Although the RAS strain pattern is described as a sensitive marker for differentiating infiltrative myocardial diseases, our study does not show that it is useful for predicting amyloidosis in patients with severe AS.

## 5. Limitations

This study had some limitations. It was a single-center study with a limited number of patients, and we analyzed only patients undergoing SAVR and did not include a TAVI population. Analysis of older patients with higher surgical risks would be useful for expanding the study group in which the association of the RAS strain pattern with amyloid infiltration could be assessed.

## 6. Conclusions

The RAS strain pattern is relatively common in low-surgical-risk, severe AS patients without biopsy and imaging-proven CA undergoing SAVR. The RAS strain pattern represents a more advanced AS stage with adverse LV remodeling and evidence of heart failure. The RAS strain pattern is strongly associated with increased LV mass and it resolves within 3 months after the removal of a pressure overload by the performance of SAVR. Nevertheless, further studies are needed to assess the prognostic value of the RAS strain pattern in AS patients undergoing either surgical or transcatheter aortic valve replacement.

## Figures and Tables

**Table 1:** Clinical, imaging, and histology characteristics of the study cohort stratified by the detection of an RAS strain pattern by GLS analysis.

Variables		All Patients (*n* = 77)	RAS Strain Pattern-Positive Patients (*n* = 14)	RAS Strain Pattern-Negative Patients (*n* = 63)	*p*-Value
Age, years		70 (62–73)	67 (63–71)	71 (62–73)	0.415
Sex, female		45 (58)	9 (64)	36 (57)	0.849
BSA, m^2^		1.9 ± 0.2	1.9 ± 0.1	1.9 ± 0.2	0.822
Comorbidities					
Hypertension		69 (90)	13 (92)	56 (88)	0.597
Dyslipidemia		61 (79)	9 (64)	52 (83)	0.128
Unobstructive CAD		30 (39)	4 (29)	26 (41)	0.378
Diabetes mellitus		12 (16)	4 (27)	8 (13)	0.139
Atrial fibrillation		2 (3)	0 (0)	2 (3)	0.499
Symptoms and functional status					
Dyspnoea		54 (70)	9 (64)	45 (71)	0.597
Chest pain		35 (47)	8 (57)	27 (45)	0.413
Syncope		8 (10)	3 (21)	5 (8)	0.135
6MWT, m		369 (300–420)	318 (284–399)	369 (332–420)	0.220
NYHA functional class	I	15 (19)	1 (7)	14 (22)	0.561 *
	II	22 (29)	5 (36)	17 (27)	
	III	37 (48)	8 (57)	29 (46)	
	IV	3 (4)	0 (0)	3 (5)	
Risk scores					
STS-PROM, %		1.6 (1.2–2.4)	1.8 (1.4–2.2)	1.6 (1.0–2.5)	0.629
EuroSCORE II, %		1.1 (0.7–1.6)	1.0 (0.8–1.5)	1.1 (0.7–1.7)	0.771
ECG parameters					
S–L voltage index, mm		31.2 ± 10	35.8 (29.5–44)	30.1 (23–37.8)	0.058
QRS duration, ms		94 (86–102)	96.0 ± (92.5–102.0)	91.0 (86.0–99.5)	0.224
Echocardiography data					
AV area index, cm^2^/m^2^		0.45 ± 0.1	0.41 ± 0.1	0.45 ± 0.1	0.163
Peak AV velocity, m/s		4.9 ± 0.6	5.3 ± 0.7	4.8 ± 0.6	**0.005**
Mean AV gradient, mmHg		54.9 (45–70)	70 ± 20	56 ± 14	**0.013**
Low gradient AS		8 (10)	1 (7)	7 (11)	0.660
IVSd, mm		12.8 ± 1.7	14.6 ± 1.1	12.4 ± 1.5	**<0.001**
LVEdd, mm		50 (47–55)	51.8 ± 4.4	51.2 ± 5.6	0.384
LVEsd, mm		32 (29–35)	34.5 ± 6	32 ± 6	0.124
E/A ratio		1.2 ± 0.5	1.4 ± 0.6	1.1 ± 0.4	**0.042**
E deceleration time, ms		245 (212–308)	223 (200–265)	251 (223–314)	0.190
Posterior wall diameter, mm		12 (10–12)	12 (12–14)	11 (10–12)	**0.001**
E/e’ septal		17 (13–21)	17.1 (16–18)	16 (13–20)	0.238
E/e’ lateral		13 (11–17)	15 (11–17)	13 (11–17)	0.804
E/e’ mean		15 (12–18)	16 ± 7	16 ± 6	0.717
LA volume index, mL/m^2^		48.6 ± 12	52 ± 14	48 ± 12	0.253
PASP, mmHg		33 (29–42)	39 ± 18	38 ± 14	0.826
RV S’, cm/s		12 (10–13)	12 (10–13)	11 (10–13)	0.771
TAPSE		22 ± 3	21 ± 3	22 ± 3	0.057
GLS, %		−18 ± 5	−14.9 ± 3	−18.7 ± 5	**0.002**
GLS >−15%		16 (21)	7 (50)	9 (14)	**0.006**
CMR data					
IVSd, mm		13.3 (12–15)	14.8 ± 1	12.9 ± 2	**0.004**
LVEdd, mm		50.4 ± 6	54 ± 4	50 ± 6	**0.006**
LVEsd, mm		33.6 ± 8	37 ± 7	33 ± 8	0.140
LVEDV, mL		136 (113–160)	149 ± 30	143 ± 348	0.265
LVESV, mL		41.5 (28–61)	56 (46–73)	36 (26–56)	0.062
LV stroke volume index, mL/m^2^		46 (42–53)	44 (43–48)	47 (41–55)	0.452
LVEF, %		69.6 (61–75)	62 ± 12	68 ± 13	**0.040**
LVEF < 50%		8 (10)	8 (20)	0 (0)	0.089
LV mass index, g/m^2^		91.3 (76–119)	125 ± 28	91 ± 32	**0.001**
RVEDV, mL		120 (108–140)	119 (103–136)	120 (109–140)	0.734
RVESV, mL		44 (36–59)	40 (30–52)	45 (37–59)	0.163
RVEF, %		62.9 (57–67)	64.7 (61–72)	61.7 (57–66)	0.119
Native T1, ms		959.2 ± 34	971 ± 36	956 ± 33	0.156
Post-contrast T1, ms		352 (327–363)	353 (332–363)	351 (327–361)	0.533
LGE prevalence		57 (74)	34 (85)	23 (62)	0.175
ECV, %		22.6 ± 3	23.4 ± 3	22.3 ± 3	0.292
ECV index, %/m^2^		12 (11–13)	13 (12–13)	12 (11–13)	0.454
Histology data (***n*** = 71)					
CVF, %		12.4 (8–22)	10.8 (7–17)	15.9 (8–23)	0.232
CVF subendocardial, %		21.8 (10–35)	17.4 (9–24)	21.8 (12–38)	0.108
Surgery					
Tissue valve		71 (92)	14 (100)	57 (91)	0.229
Mechanical valve		6 (8)	0 (0)	6 (10)	0.229
Aortic intervention		2 (3)	0 (0)	2 (3)	0.499
Serum biomarkers					
BNP, pg/L		142 (67–362)	409 (161–961)	119 (66–245)	**0.032**
Hs-Tn-I, pg/L		10 (5–18)	15 (13–29)	9 (5–18)	**0.026**
Creatinine, μmol/L		76 ± 16	73 ± 17	76 ± 16	0.491
eGFR, mL/min/1.73 m^2^		85 (69–90)	87 (71–90)	85 (70–90)	0.533

Continuous variables are presented as mean ± SD or median (interquartile range). Categorical variables are expressed as n (%). Bolded text indicates statistical significance. A, peak late velocity of trans-mitral flow; AS, aortic stenosis; AV, aortic valve; BNP, brain natriuretic peptide; BSA, body surface area; CAD, coronary artery disease; CMR, cardiovascular magnetic resonance; CVF, collagen volume fraction; E, peak early velocity of the trans-mitral flow; ECG, electrocardiogram; e’, peak early diastolic velocity of the mitral annulus displacement; eGFR, estimated glomerular filtration rate; EuroSCORE II, European System for Cardiac Operative Risk Evaluation II; ECV, extracellular volume; E/A ratio, ratio of peak velocity flow in early diastole (E wave) to peak velocity flow in late diastole (A wave); E/e’ ratio, ratio of peak velocity flow in early diastole (E wave) to peak early diastolic velocity of the mitral annulus displacement (e’ wave); GLS, global longitudinal strain; Hs-Tn-I, high-sensitivity troponin I; IVSd, interventricular septum diastolic diameter; LA, left atrium; LGE, late gadolinium enhancement; LV, left ventricular; LVEDV, left-ventricular-end diastolic volume; LVEdd, left-ventricular-end diastolic diameter; LVEF, left ventricular ejection fraction; LVESV, left-ventricular-end systolic volume; LVEsd, left-ventricular-end systolic diameter; n, number; NYHA, New York Heart Association; PASP, pulmonary artery systolic pressure measured by echocardiography; PCI, percutaneous coronary intervention; RAS strain pattern, relative apical sparing strain pattern; RV, right ventricle; RVEDV, right-ventricle-end diastolic volume; RVEF, right ventricle ejection fraction; RVESV, right-ventricle-end systolic volume; S’, right ventricle systolic excursion velocity; S_L, Sokolov–Lyon index; SD, standard deviation; STS-PROM, Society of Thoracic Surgeon’s predicted risk of mortality; TAPSE, Tricuspid Annular Plane Systolic Excursion; 6MWT, 6 min walk test. * *p*-value for comparison among NYHA I and II vs. III and IV.

**Table 2:** Baseline, 3-month, and 12-month follow-up echocardiography data from patients with an RAS strain pattern.

Echocardiography Data	Baseline (*n* = 14)	3-Month Follow-Up (*n* = 14)	12-Month Follow-Up (*n* = 14)	*p*-Value
AV area index, cm^2^/m^2^	0.4 ± 0.1	1.3 ± 0.3	1.2 ± 0.3	**<0.001**
Peak AV velocity, m/s	5.3 ± 0.7	2.2 ± 0.4	2.3 ± 0.4	**<0.001**
Mean AV gradient, mm Hg	65.3 (56.2–84.1)	9.25 (8.0–10.8)	10.4 (8.2–12.6)	**<0.001**
IVSd, mm	14.5 (14.0–15.0)	11.5 (11.0–14.0)	11.5 (11.0–12.0)	**<0.001**
LVEdd, mm	51.5 (49.0–54.5)	50.5 (48.5–53.8)	50.0 (46.5–52.0)	0.699
LVEsd, mm	34.2 ± 5.7	33.8 ± 5.9	31.9 ± 3.6	0.131
E/e’ septal	17.6 (16.1–20.1)	16.6 (11.4–18.2)	15.6 (12.7–21.5)	0.092
E/e’ lateral	14.7 (11.2–17.0)	8.43 (6.56–10.8)	8.88 (6.60–11.8)	**0.011**
E/e’ mean	17.1 ± 7.2	11.7 ± 3.7	11.9 ± 4.5	**0.004**
LA volume index, mL/m^2^	52.0 ± 13.4	45.4 ± 8.5	43.0 ± 10.8	**0.009**
RV S’, cm/s	12 (10–13)	9 (8–10)	9 (9–11)	**0.009**
GLS, %	14.9 ± 3	18.3 ± 2	19.7 ± 2	**<0.001**
RAS strain pattern, ***n*** (%)	14 (100)	0 (0)	0 (0)	–

Abbreviations are as in Table 1.

## Data Availability

The datasets are available upon request to the corresponding author.

## Abbreviations

AS	aortic stenosis
ATTR-CA	transthyretin cardiac amyloidosis
AV	aortic valve
AVA	aortic valve area
SAVR	surgical aortic valve replacement
BNP	brain natriuretic peptide
BSA	body surface area
CA	cardiac amyloidosis
CAD	coronary artery disease
CKD	chronic kidney disease
CVF	collagen volume fraction
CMR	cardiovascular magnetic resonance
ECG	electrocardiography
ECV	extracellular volume
GLS	global longitudinal strain
Hs–Tn–I	high-sensitivity troponin I
LGE	late gadolinium enhancement
LV	left ventricle
LVEF	left ventricular ejection fraction
MI	myocardial infarction
NYHA	New York Heart Association
RAS	relative apical sparing
STE	speckle-tracking echocardiography
TAVI	transcatheter aortic valve implantation
T1	T1 myocardial relaxation
